# The pVHL_172_ isoform is not a tumor suppressor and up-regulates a subset of pro-tumorigenic genes including *TGFB1* and *MMP13*

**DOI:** 10.18632/oncotarget.18376

**Published:** 2017-06-06

**Authors:** Pauline Hascoet, Franck Chesnel, Florence Jouan, Cathy Le Goff, Anne Couturier, Eric Darrigrand, Fabrice Mahe, Nathalie Rioux-Leclercq, Xavier Le Goff, Yannick Arlot-Bonnemains

**Affiliations:** ^1^ CNRS, UMR 6290 IGDR, Université Rennes 1, BIOSIT, Rennes, France; ^2^ IRMAR, Université Rennes 1, Rennes, France; ^3^ CHU Rennes, Service d’Anatomo-Pathologie, Rennes, France

**Keywords:** von Hippel Lindau, tumor suppressor, human kidney cells, TGF-β signaling, metalloprotease

## Abstract

The von Hippel-Lindau (*VHL*) tumor suppressor gene is often deleted or mutated in ccRCC (clear cell renal cell carcinoma) producing a non-functional protein. The gene encodes two mRNA, and three protein isoforms (pVHL_213_, pVHL_160_ and pVHL_172_). The pVHL protein is part of an E3 ligase complex involved in the ubiquitination and proteasomal degradation of different proteins, particularly hypoxia inducible factors (HIF) that drive the transcription of genes involved in the regulation of cell proliferation, angiogenesis or extracellular matrix remodelling. Other non-canonical (HIF-independent) pVHL functions have been described. A recent work reported the expression of the uncharacterized protein isoform pVHL_172_ which is translated from the variant 2 by alternative splicing of the exon 2. This splice variant is sometimes enriched in the ccRCCs and the protein has been identified in the respective samples of ccRCCs and different renal cell lines. Functional studies on pVHL have only concerned the pVHL_213_ and pVHL_160_ isoforms, but no function was assigned to pVHL_172_. Here we show that pVHL_172_ stable expression in renal cancer cells does not regulate the level of HIF, exacerbates tumorigenicity when 786-O-pVHL_172_ cells were xenografted in mice. The pVHL_172_-induced tumors developed a sarcomatoid phenotype. Moreover, pVHL_172_ expression was shown to up regulate a subset of pro-tumorigenic genes including *TGFB1*, *MMP1* and *MMP13*. In summary we identified that pVHL_172_ is not a tumor suppressor. Furthermore our findings suggest an antagonistic function of this pVHL isoform in the HIF-independent aggressiveness of renal tumors compared to pVHL_213_.

## INTRODUCTION

The human von Hippel-Lindau (*VHL)* tumor suppressor gene encodes three different protein isoforms. The pVHL_213_ (213 amino acids) and pVHL_160_ (160 amino acids; absence of 54 NH_2_-terminal amino acids due to the use of an internal start site) isoforms are translated from the full length mRNA [1, 2], whereas pVHL_172_ (172 amino acids) is encoded by an mRNA in which exon 2 is excluded by alternative splicing [3, 4]. *In vivo*, pVHL_213_ and pVHL_160_ exert equivalent functions. Specifically, they show tumor suppressor activity in a clear cell Renal Cell Carcinoma (ccRCC) cell xenograft model [2, 5]. The pVHL_213_ is the substrate recognition subunit of an E3 ubiquitin ligase complex that also includes elongins B and C, cullin-2 and RBX1. This complex targets hypoxia-inducible factor alpha (HIF-α) for proteasomal degradation. Inactivation of pVHL stabilizes HIF-α that heterodimerizes with HIF-β and translocates in the nucleus to activate the transcription of many genes involved in the hypoxic response and other pro-tumorigenic processes [6]. Loss of pVHL function is crucial in different pathologies and has been largely studied in ccRCC, the most common kidney cancer type. More than 80% of sporadic ccRCC show pVHL deficiency [4, 7]. Loss of *VHL* function by deletion, mutation or promoter hypermethylation contributes to ccRCC initiation by promoting HIF-dependent overproduction of proangiogenic factors, including VEGF and PDGF.

Moreover, other non-canonical (*i.e.*, HIF-independent) pVHL functions have been described, such as regulation of cell-cell interaction, matrix signaling and adhesion [8], which may also contribute to pVHL tumor suppressor activity [9]. For instance, extracellular fibronectin matrix is defective in renal carcinoma cells lacking pVHL, suggesting a direct pVHL role in fibronectin matrix formation. Loss of pVHL in ccRCC has also been associated with modulation of TGFB1 expression and poor prognosis [10, 11]. Elevated levels of TGFB1 in serum samples from patients with ccRCC are correlated with unfavorable outcome and ccRCC microenvironment is TGFB1-rich. TGFB binding to and activation of the TGFB receptors TGFBRI and TGFBRII at the plasma membrane activates the TGFB signaling pathway. Activated TGFBRI phosphorylates SMAD2 and/or SMAD3 that form a complex with SMAD4 to regulate transcription of many target genes [12].

The pVHL_172_ isoform was recently differentially detected in cells and tumor tissues and its putative effect on tumor progression needs to be investigated. Importantly, it has been reported that some *VHL* mutations may favor the expression of VHL variant 2 in ccRCC [4, 13, 14]. To determine how this pVHL_172_ enrichment in some tumoral cell may affect tumor development, we stably expressed pVHL_172_ in 786-O cells (derived from a human primary clear cell renal adenocarcinoma: pVHL null cells). Mice xenografted with pVHL_172_-expressing 786-O cells developed tumors with more extended sarcomatoid phenotype than tumors derived from parental 786-O cells. Expression of pVHL_172_ stimulated TGFB signaling and upregulation of the metalloproteases MMP13 and MMP1, while pVHL_213_ expression downregulated these genes. Our study unravels a pVHL_172_ positive role in tumor progression, suggesting that the expression balance of the different pVHL isoforms has a critical role in ccRCC initiation and progression.

## RESULTS

### The expression of pVHL_172_ modifies behavior of 786-O cells

**Figure 1:**
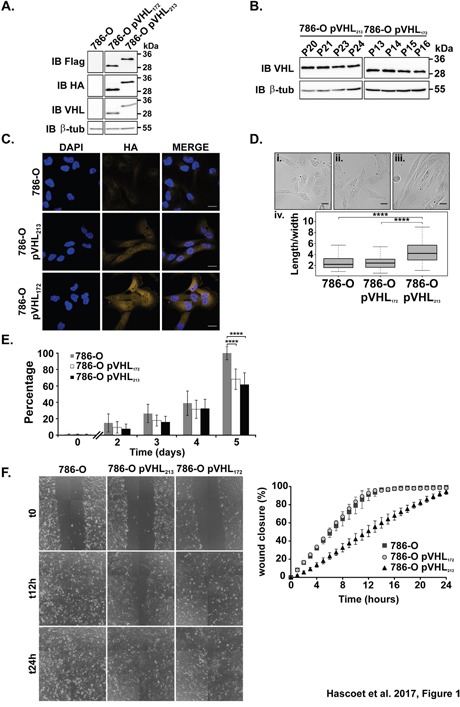
Analysis of the phenotypes of the cells expressing pVHL_172_ or pVHL_213_ **(A)** pVHL expression in 786-O, 786-O-pVHL_172_ and 786-O-pVHL_213_ cells assessed by immunoblotting with the indicated antibodies. **(B)** pVHL expression at different passages in the stable 786-O-pVHL_172_ and 786-O-pVHL_213_ cell lines assessed by immunoblotting. **(C)** pVHL expression analyzed by immunocytochemistry with an anti-HA antibody in 786-O (upper panels), 786-O-pVHL_213_ (middle panels) and 786-O-pVHL_172_ cells (lower panels). Nuclei were stained with DAPI (scale bar: 25μm). **(D)** The length and width of 786-O cells (i), 786-O-pVHL_172_ (ii) and 786-O-pVHL_213_ (iii) cells were measured and the length/width ratio (iv) was calculated (n=100/each, ****: p<0.0001, Mann-Withney test). Scale bar: 25μm. **(E)** Proliferation of 786-O, 786-O-pVHL_172_ and 786-O-pVHL_213_ cells was assessed using the PrestoBlue™ assay. Values were normalized to the mean 786-O cell number at day 5 (mean±s.d. of three independent experiments with eight independent samples; ****: p<0.0001, Mann-Withney test performed at day 5). **(F)** Analysis of cell migration by wound healing assay. Results were expressed as the percentage of wound closure at the indicated time points (mean±s.d. of three independent samples representative of three independent experiments).

The vhl gene encodes two mRNA variants and three different protein isoforms (Supplementary Figure 1). The expression of the variant 2 mRNA of the *vhl* gene was evidenced and the presence of the corresponding protein was detected in different cells lines and in renal tumor tissues [3]. Whereas pVHL_213_ was characterized as a tumor suppressor gene, pVHL_172_ function has never been investigated yet. In order to investigate this pVHL_172_ function we generated stably transfected cells lines with pVHL_172_ (or pVHL213 as a control). The level of pVHL expression was stable over several passages in both cell line (Figure 1A, 1B). Analysis of the half-life of the proteins showed a slight decrease in pVHL_213_ expression after 6 hours of incubation with cycloheximide (CHX, Supplementary Figure 2A). Conversely, pVHL_172_ expression remained stable, whereas cyclin D (CCD1) expression (used as positive control) strongly decreased after 30 min of CHX incubation, in agreement with published results [15]. Anti-HA immunostaining showed that pVHL was broadly expressed in all cells in pVHL_172_-expressing and pVHL_213_-expressing 786-O cells, but not in parental 786-O cells (Figure 1C). pVHL_172_ expressing 786-O cells are more spread than the pVHL_213_-expressing 786-O cells (Figure 1D) as confirmed by tubulin network labelling (Supplementary Figure 2B). The cell width and length were measured in the three cell lines (n=100 cells). The length/width ratio of 786-O and 786-O-pVHL_172_ cells was comparable (2.7 and 2.6, respectively) (Figure 1D.i and 1D.ii), whereas it was significantly higher in 786-O-pVHL_213_ cells (4.6) (Figure 1D iii. and 1D iv.).

We then performed functional assays to determine whether the expression of pVHL_172_ modified cell behavior compared to the cells expressing pVHL_213_. The cell proliferation was significantly slowed down (at day 5) in cells that expressed pVHL_172_ or pVHL_213_ compared with parental 786-O cells (no pVHL expression) (*p* = 1.24 × 10^−13^ and, *p* = 4.341 × 10 ^−13^ respectively) (Figure 1E). Analysis of cell motility by using a wound-healing assay showed complete closure of the wound after 12 hours in 786-O and 786-O- pVHL_172_ cells. Conversely, wound closure was not complete in 786-O-pVHL_213_ cells even after 24 hours (Figure 1F). This was caused by cell migration inhibition and not the result of both cell migration and proliferation defects because identical results were obtained in the presence of mitomycin C to inhibit cell proliferation (Supplementary Figure 2C). The expression of pVHL_172_ conferred to the 786-O cells behavior modifications related occasionally to the 786-O-pVHL_213_ cells or to the 786-O and this prompted us to consider the tumor suppressor gene function of this isoform *in vivo*.

### The expression of pVHL_172_ induces an oncogenic phenotype in 786-O cells and does not act as a tumor suppressor in mice

**Figure 2:**
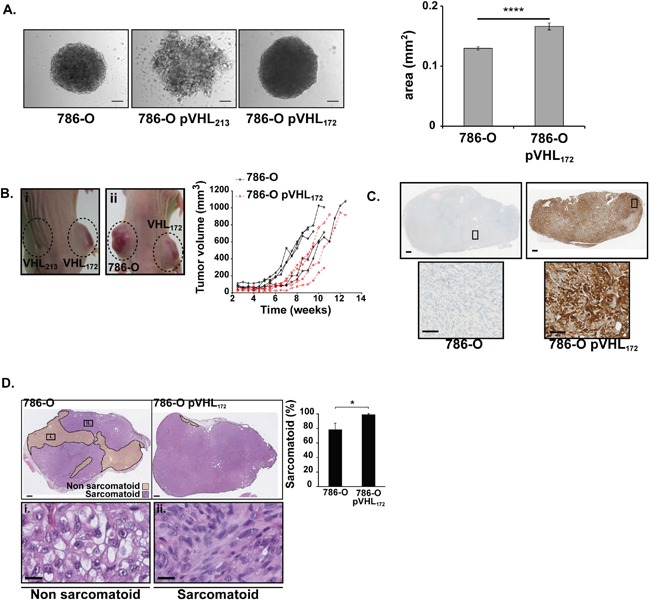
Tumorigenic effect 786-O-pVHL_172_ cell-derived tumors compared with 786-O cell-derived tumors **(A)** Morphology of 786-O, 786-O-pVHL_213_ and 786-O pVHL_172_ spheroids (left panels). Spheroid areas from 768-O and 786-O-pVHL_172_ cells were measured with the ImageJ sofware (right histogram, mean±sem, n=58 and n=45 respectively; Mann-Withney test, ****: p<0.0001). Scale bars: 100 μm. **(B)** External morphology of tumors obtained by xenografting 786-O-pVHL_172_ de mice (i-left flank and ii-right flank), 786-O-pVHL_213_ (i-right flank) or 786-O cells (ii-left flank) in nude mice. Growth kinetics of 786-O and 786-O-pVHL_172_ derived tumors determined by measuring the tumor volumes using a caliper (n=5 tumors per cell type). **(C)** pVHL_172_ expression was controlled by immunohistochemical staining with an anti-HA antibody in 786-O-pVHL_172_ and 786-O cell-derived tumors (upper panels; scale bar: 500μm). A 20X magnification is shown in the lower panels (scale bar: 100μm). **(D)** Sarcomatoid and non-sarcomatoid areas were delimitated following HE staining of 786-O and 786-O-pVHL_172_ cell-derived tumor sections (upper panels, scale bar: 500μm). Inserts in a representative 786-O tumor section show the gradual progression of the tumor phenotype from (i) non-sarcomatoid morphology to (ii) a tissue with sarcomatoid component (scale bar: 20μm). The histogram shows the percentage of sarcomatoid areas relative to the whole tumor section (mean ± s.d., n=5 tumors/cell type; Mann-Whitney test: p<0.05).

A 3D *in vitro* cell culture model assay was adopted as a preclinical model tool for studying tumor cell behaviour. A spheroid formation assay was made to determine whether pVHL_172_ expression in cells could modify the properties of the 786-O cells to aggregate. Cultivating the cells on a non-adherent surface induced the formation of spheroids in the case of 786-O cells (mean size 0.12 mm^2^, n=58) and the 786-O-pVHL_172_ cells (mean size 0.16 mm^2^, n=45) (Figure 2A). In contrast, the 786-O-pVHL_213_ cells did only form cell clumps. Interestingly, the expression of pVHL_172_ in cells induced spheroids formation which size was significantly larger (Figure 2A, right panel; p-value=3.805 10^-5^). This assay demonstrated that the expression of pVHL_172_ did not suppress cell aggregation as observed with the expression of pVHL_213_. In order to further explore this property, we used a heterotopic cell line derived xenograft model. Ten millions of 786-O, 786-O-pVHL_213_ or 786-O- pVHL_172_ cells were injected subcutaneously in nude mice. The injection of 786-O-pVHL_213_ cells failed to induce tumor in mice (n=8) after 16 weeks. The volume of each tumor growing from 786-O or 786-O-pVHL_172_ cells was monitored twice a week (Supplementary Table 1). The tumors (75 mm^3^) were detectable after three and seven weeks for 786-O cells and 786-O pVHL_172_ cells, respectively. The size of the tumors increased up to the end of the experiment (Figure 2B). We found no evidence of metastases formation. We fitted the experimental values of individual growth for each tumor using a second degree polynomial interpolation. The average “*start”* value (defining when the tumor was measurable) was 3.64±0.97 weeks for 786-O tumors and 4.42±0.65 weeks for 786-O-pVHL_172_ tumors, suggesting a delayed growth induction for 786-O-pVHL_172_ cell-derived tumors but that was statistically non significant. During the effective tumor growth, the asymptotic speed of growth of 786-O-pVHL_172_ tumors was significantly higher (4.20±0.78 *vs* 3.12±0.65 for 786-O tumors; t-test: *p=0.044*). When analysing later time points from weeks 5 to 10, the growth rate of 786-O-pVHL_172_ tumors was higher than that of 786-O tumors (860 mm^3^ and 551 mm^3^ at 10.5 weeks for 786-O-pVHL_172_ and 786-O respectively). Expression of the pVHL_172_ performed on the harvested 786-O-pVHL_172_ cell-derived tumors revealed the presence of pVHL over time, (Figure 2C). Thus pVHL_172_ expression slightly delayed tumor growth induction, but then increased tumor growth speed. This result indicated that opposite to the long pVHL_213_ isoform, pVHL_172_ expression in kidney cancer cells did not exert a tumor suppressor function but rather induces dissimilar morphological modifications in spheroids as in tumor compared to the spheroids or tumors generated with the parental 786-O cells.

The striking and consistent differences in the morphology between spheroids and tumors expressing pVHL172 prompted us to examine the molecular a biochemical parameters in the structures. A hallmark of renal tumors is the sarcomatoid changes considered as terminally dedifferentiated status. We performed hematoxylin and eosin (HE) staining on sections of 786-O and 786-O-pVHL_172_ tumors and spheroids (Figure 2D, Supplementary Figure 3). We observed cells with large and clear cytoplasm in the non sarcomatoid regions while the sarcomatoid phenotype was characterised by the presence of elongated, spindle-shaped, cells (Figure 2D). Interestingly, the sarcomatoid component was significantly higher in 786-O-pVHL_172_ derived tumors as it represented 99.1±1.5% in those tumor sections *vs* 78.6±17.3% in 786-O cell-derived tumor sections (*p<0.05*) (Figure 2D). An identical observation was made with HE-stained spheroids (Supplementary Figure 3). Delimitation and measurement of necrotic areas in HE-stained tumor sections did not highlight more than 5% of necrotic areas in 786-O-pVHL_172_ tumors (data not shown). In summary to these observations, we concluded that the expression of pVHL_172_ rather promoted a higher percentage of the sarcomatoid phenotype in tumors that is correlated to a poor prognostic factor in kidney cancer. Collectively, all the results indicated that expression of pVHL_172_ exacerbates molecular pathways previously observed in tumoral VHL null cells (*i.e.* 786-O parental cells).

### pVHL_172_ expression in cells induces specific metalloproteases over-expression

**Figure 3:**
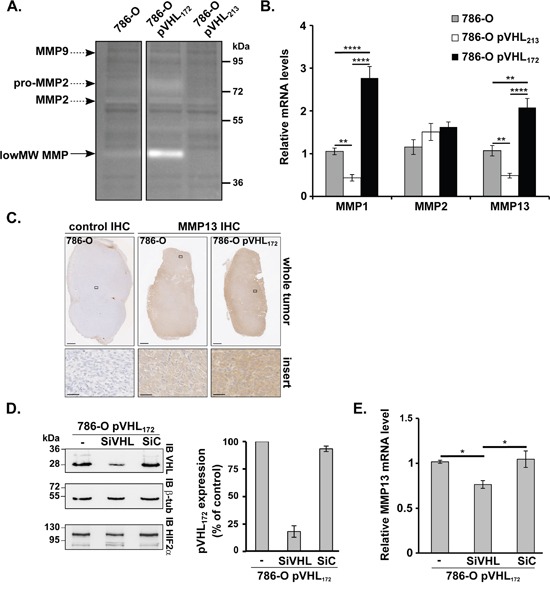
pVHL_172_ promotes *MMP13* upregulation **(A)** Zymography assay performed using gelatin-containing gels and 786-O, 786-O-pVHL_172_ and 786-O-pVHL_213_ protein extracts. Arrows indicate the digestion areas corresponding to the different pro-MMPs or MMPs, according to their molecular weights. Low MW MMP: low molecular weight MMPs (possibly MMP1, -3, -8, -13, or -23). **(B)** RT-qPCR assays using reverse-transcribed RNAs extracted from 786-O, 786-O-pVHL_213_ and 786-O-pVHL_172_ cells were performed to monitor the expression of *MMP1*, *MMP2* and *MMP13*. **(C)** MMP13 expression analyzed by immunohistochemistry in whole 786-O and 786-O-pVHL_172_ cell-derived tumors (upper panels; scale bar: 1 mm). A 40X magnification is shown in the lower panels (scale bar: 50 μm). Control: secondary antibody alone (left panels). **(D)** Efficiency of siRNA-mediated pVHL_172_ downregulation assessed by immunoblotting with anti-pVHL_172_ and -β-tubulin (control) antibodies in 786-O-pVHL_172_ cells harvested 72 hours after transfection with siRNAs against *VHL* RNA variant 2 (SiVHL), control siRNA (SiC) or transfection reagent alone (-). The histogram shows the quantification of the results (mean ±SEM; n=4); pVHL_172_ expression in control cells (-) was set to 100. **(E)** RT-qPCR analysis of *MMP13* expression in 786-O-pVHL_172_ cells transfected with SiVHL, SiC or transfection reagent alone (-). RNA levels were normalized to *GAPDH* and *RPLPO* and the expression level in control 786-O-pVHL_172_ cells (-) was set to 1. (n=4). *: p<0.05; **: p<0.01; ***: p<0.001; ****: p<0.0001, Mann-Whitney test.

Proteolytic degradation of the extracellular matrix is considered as essential step for invasion and metastasis by cancer cells and malignant transformation has been often shown to associate with alteration of several metalloproteases [16]. MMP expression and activity in the three cell lines were assessed with two different zymography assays (Figure 3A).

The gelatinase assay in non-reducing conditions was originally used to check MMP2 and MMP9 activity levels. Extracts from 786-O cells induced digestion areas at ~95, ~80 and ~65 kDa that corresponded to the molecular weights of pro-MMP2/MMP2 and pro-MMP9/MMP9, respectively (Figure 3A). A digestion area at the pro-MMP2 level was observed with 786-O-pVHL_172_ cell extracts, whereas no or modest degradation was observed with 786-O-pVHL_213_ or 786-O cell extracts. Moreover, a stronger degradation area at around ~45 kDa was detected in 786-O-pVHL_172_ and 786-O cell extracts (to a lesser extent) (Figure 3A, low MW MMP). These results suggest that pVHL_172_ expression may promote the expression and/or activity of low MW MMPs, which include MMP1, -3, -8, -13 or -23. A second zymography assay with collagen type I as substrate has confirmed this result (data not shown). As MMP1, MMP8 and MMP13 are all known as both gelatinases and collagenases (collagen I), we investigated whether these MMPs may be target of pVHL_172_ expression. Among all selected MMPs, a q-PCR analysis using RNA extracted from the three cell lines showed that only *MMP1* and *MMP13* mRNAs were upregulated in 786-O-pVHL_172_ cells compared with 786-O and both were down-regulated in 786-O-pVHL_213_ cells. The MMP2 expression was not significantly changed in the three cell lines (Figure 3B). No difference in *MMP9* mRNA expression could be detected in any of the three cell lines (data not shown). The immunohistochemistry analysis of MMP13 expression in tumors derived from 786-O-pVHL_172_ cells and parental 786-O cells (control) showed a stronger MMP13 signal in pVHL_172_-expressing tumors than in controls (Figure 3C). None of the tumor expressing pVHL_172_ showed an increased signal for MMP1. Thus we focused our analysis on MMP13. To confirm that *MMP13* upregulation was pVHL_172_-dependent, pVHL_172_ was knocked down in 786-O-pVHL_172_ cells by using a specific VHL_172_ siRNA (SiVHL). Upon SiVHL transfection, pVHL_172_ expression was reduced by 82% compared with non-transfected cells and cells transfected with control siRNA (SiC) (Figure 3D). The amount of MMP13 mRNA did not change between 786-O and control SiRNA transfected cells. In contrast, the relative amount of *MMP13* mRNA was significantly reduced in pVHL_172_-depleted cells (Figure 3E), indicating that *MMP13* expression may be regulated by the expression of pVHL_172_. Altogether, these results show that conversely to pVHL_213_, pVHL_172_ upregulates directly or indirectly the collagenase MMP13.

### pVHL_172_ does not down-regulate HIF-2α expression even if it still participates to the E3 ubiquitin ligase complex

**Figure 4:**
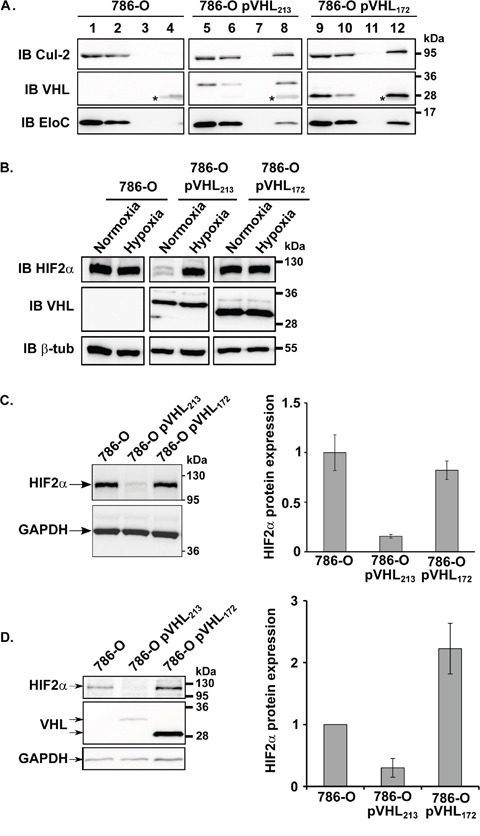
pVHL_172_ is part of an E3 ubiquitin ligase complex, but is not involved in HIF-2α down-regulation **(A)** pVHL, cullin-2 and elongin C expression in cell lysates (1, 5, 9), unbound (2, 6, 10), wash (3, 7, 11) and Flag-immunoprecipitated (4, 8, 12) fractions of 786-O, 786-O-pVHL_213_ and 786-O-pVHL_172_ cells was assessed by immunoblotting (*: IgG light chain). **(B)** HIF-2α protein expression level was evaluated by immunoblotting in 786-O, 786-O-pVHL_213_ and 786-O-pVHL_172_ cells in normoxia or hypoxia conditions. β-Tubulin was used as a loading control to quantify the level of HIF-2α. **(C-D)** HIF-2α expression level was evaluated by immunoblotting in 786-O, 786-O-pVHL_213_ and 786-O-pVHL_172_ spheroids. GAPDH was used as a loading control to quantify the level of HIF2α.

In recent years, our understanding of pVHL function has broadened to include several HIF-independent features. Hypoxia and HIFs can induce tumor cell invasion and degradation of the extracellular matrix *via* various mechanisms including upregulation of matrix metalloproteinases-1 and -2 [17, 18]. In chondrocytes, HIF-2α directly induces the expression of genes encoding catabolic factors, including matrix metalloproteinases (*MMP1*, *MMP9*, *MMP12* and *MMP13*) [19]. We wonder whether pVHL_172_ might regulate the expression of the MMPs in a HIF-dependent way. HIF amount in cells is regulated by the E3 ligase complex in which pVHL protein assembles with associated proteins: elongins B and C, cullin-2, and Rbx. This E3 ubiquitin ligase targets the α−subunits of hypoxia-inducible factor (HIFα) for oxygen-dependent degradation. HIF-2α is stabilized by hypoxia or mutations of pVHL. In pVHL_213_, exon 2-encoded residues 114–154 are mostly hydrophobic and are hypothesized to play a role in substrate protein recognition, although *in vitro* experiments have revealed that they might not be required for pVHL binding to HIFα [20]. In order to understand the absence of tumor suppressor function for pVHL_172_, we investigated whether HIF-2α is regulated by this isoform by first studying the belonging of pVHL_172_ to the same E3 ligase complex. Total protein extracts from 786-O-pVHL_172_, 786-O-pVHL_213_ and parental 786-O cells were analysed by immuno-precipitation assay (Figure 4A). Elongin C and cullin-2 co-immunoprecipitated with Flag-tagged pVHL_213_, as expected, and also with Flag-tagged pVHL_172_ (Figure 4A). Conversely, none of these proteins were found in the fraction immunoprecipitated from 786-O cells (Figure 4A). We secondly confirmed that the protein HIF-2α was not degraded in cells expressing pVHL_172_ compared to the 786-O-pVHL_213_ cells and was similar to that of the parental 786-O cell line under normoxia or hypoxia conditions (Figure 4B). Likewise, HIF-2α was not degraded in spheroids resulting from 786-O expressing or not pVHL_172_; it was even notably higher in spheroids expressing pVHL_172_ compared to the 786-O-derived spheroids (Figure 4C-4D). These results highlighted that pVHL_172_ does not regulate HIF stability but still retain the capacity to form a E3 ubiquitin ligase complex. As the 786-0 and the 786-O-pVHL_172_ shared the same HIF-2α status, we speculated that exacerbation of tumorigenic features in cell lines which expressed pVHL_172_, may therefore be regulated by alternative HIF-independent pathway(s).

### pVHL_172_ regulates the expression of MMPs via the TGFB signalling

**Figure 5:**
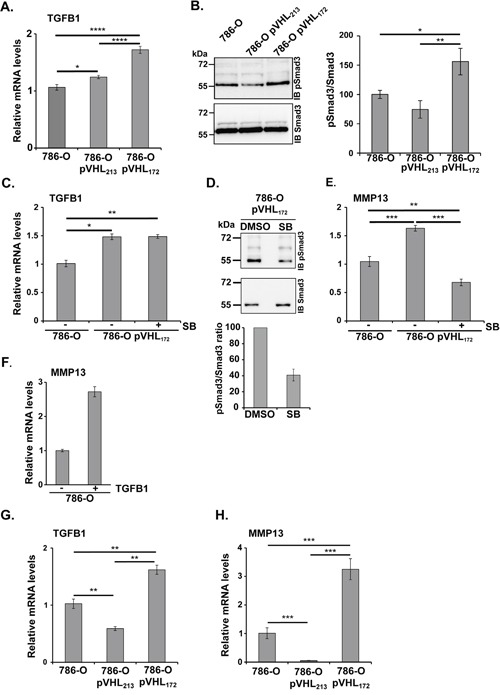
pVHL_172_ promotes TGFB and MMP13 upregulation **(A)** RT-qPCR analysis of *TGFB* expression in 786-O, 786-O-pVHL_172_ and 786-O-pVHL_213_ cells. *GADPH* was used as control. (*: p<0.05; ****: p<0.0001; ANOVA test). **(B)** Phosphorylated SMAD3 (upper panel) and total SMAD3 levels (lower panel) in 786-O, 786-O-pVHL_172_ and 786-O-pVHL_213_ cells were assessed by western blot analysis. Histogram on the right shows the quantification of the phosphorylated SMAD3/total SMAD3 ratio in 786-O, 786-O-pVHL_213_ and 786-O- pVHL_172_ cells. (*: p<0.05; **: p<0.01; ANOVA test). **(C)** RT-qPCR analysis of *TGFB* mRNA expression in 786-O and 786-O-pVHL_172_ cells incubated with SB431542 (SB) or with DMSO (control). Control *TGFB* mRNA levels (DMSO-treated 786-O cells) was set to 1. **(D)** Phosphorylated SMAD3 (upper panel) and total SMAD3 (lower panel) level was assessed by immunoblotting in 786-O-pVHL_172_ cells treated with SB431542 (SB), a TGFB receptor inhibitor, or with DMSO. The phosphorylated SMAD3/total SMAD3 ratios are shown in the histogram; the ratio of control cells (DMSO) was set to 100. **(E)** RT-qPCR analysis of MMP13 mRNA expression in 786-O and 786-O-pVHL_172_ cells incubated with SB431542 (SB) or with DMSO (control). Control mRNA levels (DMSO-treated 786-O cells) was set to 1. **(F)** RT-qPCR analysis of *MMP13* expression in 786-O cells incubated with the TGFB (5 ng/ml). (**: p<0.01; ***: p<0.001; Mann-Whitney test). **(G-H)** Rt-qPCR analysis of *TGFB (F) and MMP13 (G)* expression in spheroids of 786-O, 786-O-pVHL_172_ and 786-O-pVHL_213_ cells. (**: p<0.01; ***: p<0.001; Mann-Whitney test).

The cytokine Transforming Growth Factor-b (TGF-b) has been extensively studied in tumor biology and is believed to serve a variety of functions in tumor progression. Increased expression of the MMPs (MMP13 in particular) was also demonstrated to be stimulated by TGFB1 [21]. We analysed the *TGFB1* mRNA by RT-qPCR and observed a significant increase in 786-0-pVHL_172_ cells compared with parental 786-O cells (Figure 5A). To determine whether this increase was accompanied by *TGFB* signalling activation, total SMAD3 and phosphorylated SMAD3 (pSMAD3) levels were assessed by western blotting. Compared with parental 786-O cells, pSMAD3 was significantly increased in 786-O-pVHL_172_ cells and reduced in 786-O- pVHL_213_ cells (Figure 5B). Moreover, a 24-hour incubation with SB431542, a TGFBR1 inhibitor, did not affect *TGFB1* mRNA up regulation (Figure 5C) but markedly reduced pSMAD3 level in 786-O-pVHL_172_ cells (Figure 5D). As pVHL_172_ was suspected to control the MMPs levels via the TGFB signalling, the effect of this TGFBR1 inhibitor on *MMP13* expression in 786-O-pVHL_172_ cells was assessed by RT-qPCR analysis. The experiment showed that TGFB signalling inhibition reduced the expression of *MMP13* (Figure 5E). Conversely, when incubating the 786-O cells in the presence of TGFB, we observed a two-fold increase of MMP13 expression (Figure 5F). Having demonstrated that pVHL_172_ induced the expression of MMP13 in cells in a TGFB-dependent manner, we further tested the expression of both MMP13 and TGFB *in vitro* in the 3D spheroid models. In spheroids expressing pVHL_172_, we first observed an increase of TGFB mRNA (Figure 5G) associated with an increase of MMP13 transcript expression (Figure 5H). These findings clearly demonstrate the ability of pVHL_172_ to positively regulate MMP-13 expression via TGFB signaling in 786-O-pVHL_172_ cells and suggest that Smad/pSmad may participate in this (TGFB) regulatory pathway.

## DISCUSSION

In this work, we show that contrarily to the long isoform pVHL_213_, pVHL_172_ is not a tumor suppressor but rather exacerbates renal tumor phenotype. Specifically, we demonstrated in our model that: (a) pVHL_172_ expression in 786-O cells does not modify the cell phenotype, but reduces cell proliferation, (b) cultured on non-adherent surface, pVHL_172_-expressing cells generates larger spheroids (c) when xenografted in mice, pVHL_172_-expressing 786-O cells produce tumors with a higher sarcomatoid component compared with tumors derived from parental cells (d) the function of pVHL_172_ does not depend upon HIF-2α regulation. Our results strongly suggest that the tumor phenotype could be in part dependent on pVHL_172_-mediated upregulation of *TGFB1* and of some metalloproteases.

Loss of the *VHL* gene plays an important role in the development of sporadic or hereditary ccRCC in some patients with VHL disease [4]. Here, we show that in contrast to pVHL_213_, expression of pVHL_172_ in 786-O cells does not inhibit tumor growth and promotes a more dedifferentiated tumoral phenotype compared to the tumors induced with parental 786-O cells.

Literature data indicate that pVHL is involved in the fine regulation of cell proliferation, survival and angiogenesis by controlling the stability of hypoxia inducible transcription factors α (HIF-1α, HIF-2α and HIF-3α, collectively HIF-α) [22]. pVHL interact with elongin C (*via* their α domain), elongin B and cullin-2 in an E3 ubiquitin ligase complex that targets substrates for degradation by the proteasome. Interestingly, like pVHL_213_, pVHL_172_ also can interact in the E3 ligase complex by association with elongins B & C and cullin-2. However, it does not have any effect on HIF stability in normoxic conditions (Figure 4). Moreover, overexpressed pVHL_172_ does not compete with pVHL_213_ for the binding to other E3 ligase components and consequently, does not antagonize pVHL_213_-mediated HIF regulation (Supplementary Figure 4). This suggests that pVHL_172_, as part of the cullin-2/elongin B/elongin C/RBX1 complex, could act as a new E3 ligase substrate recognition component, unrelated to hypoxia signaling. The absence of part of the VHL β domain in pVHL_172_ might alter the sheet number and position in the structure and this could modify some protein functional activities such as specificity of substrate recognition.

We demonstrated that the pVHL_172_ isoform has an opposite effect on renal tumor progression compared with full-length pVHL_213_. pVHL_213_-expressing tumor cells show reduced cell proliferation, motility and invasion *in vitro* [23]. In contrast, motility and invasiveness of pVHL_172_-expressing cells and parental 786-O cells are undistinguishable. Only cell proliferation was slower in 786-O-pVHL_172_ cells, possibly explaining the slightly slower initial growth of tumors derived from 786-O pVHL_172_ cells compared with 786-O cells.

Our study also evidences that pVHL_172_ expression correlates with a higher proportion of sarcomatoid areas in tumor sections. We also observe an immature vasculature in the tumors expressing pVHL_172_ (Supplementary Figure 5). As previously mentioned, tumor vessels remain immature and lack the tight association between mural cells and endothelial tubes dependent on the excessive VEGF synthesis [24, 25]. Moreover, sarcomatoid differentiation usually arises within high-grade ccRCC representing a late step in the progression of this tumor type [26]. This phenotype could result from HIF-mediated transcriptional activation of VEGF and many other pro-angiogenic genes in a severe hypoxic environment [27]. However, besides HIF-2α stabilization in pVHL_172_-expressing cells, impaired angiogenesis due to overexpression of MMP1 and MMP13, which have been implicated in vascular regression [18], could also contribute to the increase in immature vasculature. Other mechanisms cannot be excluded. Several lines of evidences suggest that the function of VHL is likely to extend beyond its crucial role in oxygen signal transduction, and the loss of its function may result in deregulation of several signalling pathways that play key roles in biological processes such as cell proliferation, cell survival, cell invasion and metastasis [28].

High percentage of sarcomatoid transformation has been associated with worse outcome in patients with ccRCC [29]. The sarcomatoid component of RCC has been correlated with TGFB pathway activity [30, 31]. TGFB is upregulated in renal carcinoma and its overexpression is associated with Fuhrman grade III and IV cancers and the presence of metastases [32, 33]. Here, we found that in pVHL_172_-expressing cells, TGFB1 is upregulated and this could also promote cell invasiveness. Conversely, pVHL_213_ negatively regulates TGFB expression at both the transcriptional and protein levels [10]. Among the different targets of TGFB signaling, SMAD- and MAPK-dependent upregulation of the matrix metalloproteinase MMP13 has been reported [34]. Moreover, different MMPs are upregulated in ccRCC and their expression correlates with advanced tumor grades, reduced cell survival and the presence of metastases [35, 36]. Particularly elevated levels of MMP2 and MMP9 were found in various cancers as kidney and are associated to a poor prognosis [37]. Recently, Sassano *et al.* also reported that high MMP-13 expression correlated significantly with adverse overall survival, while MMP-1 independently did not show any significant correlation with survival [38]. Moreover, MMP13 level has been shown to be regulated by TGFB induced signals [39].

Here, we show that the TGFB pathway downstream effector SMAD3 is consistently more phosphorylated in pVHL_172_ expressing cells, indicating hyper activation of the TGFB canonical pathway associated with an over-expression of *MMP13* and *MMP1* genes. Incubation of 786-O-pVHL_172_ cells with an inhibitor of TGFB signalling shows that the expression of *MMP13* is controlled by pVHL_172_ through TGFB-dependent mechanisms. Consistently, *MMP13* expression was also increased in 786-0-pVHL172 cell-derived spheroids and tumors compared with 786-O cell-derived spheroids and tumors. The molecular mechanisms by which pVHL_172_ regulates TGFB and MMPs expression appear to be HIF-2α independent. Previous proteomic analysis reported SETBD1and TCF25 as interactors for pVHL (Δ114-154) [40]. These proteins were recognized as co transcriptional repressor for respectively Runx2 and SRF, both involved in the transcriptional co-regulation for genes like MMP13 or MMP1 [41, 42]. We made the hypothesis that in these two cases, pVHL172 by interacting with SETDB1 or TCF25, could interfere with their inhibitory effect on Runx2 or SRF. The precise cellular mechanisms by which pVHL_172_ regulates TGFB and MMPs expression remain however to be clarified.

In conclusion, the von Hippel Lindau isoform pVHL_172_ is not a tumor suppressor protein. Moreover, it does not simply behave as a dominant negative form of pVHL_213_ as its expression in 786-O cells triggers the formation of higher sarcomatoid xenograft tumors compared with parental 786-O cells that do not express pVHL. Our findings suggest a critical role of pVHL_172_ in activating a subset of pro-tumorigenic genes, including *TGFB* and *MMP13.* Future work will be focused on characterization of the mechanistic links between pVHL_172_ HIF-independent functions *via* TGFB and/or the E3-ligase complex in cancer cell invasion and metastasis formation to consider new therapeutic strategies in the ccRCC. Our data support that the presence of pVHL_172_ in cells may provide a growth advantage to affect the tumor progression and the physiological impact of the balance of expression of pVHL_213_ and /or pVHL_172_ remain to be explored.

## MATERIALS AND METHODS

### Plasmids

The plasmids pcDNA3.1-*P*_PGK_-FlagHA-VHL_213_ and -VHL_172_ were generated as follows. The ORF of VHL variant 1 (excised from pCMV2c-VHL_213_, a generous gift from Dr A. Buchberger, Würzburg, Germany) or variant 2 (AA 2-172; GenBank NM_198156) was subcloned in pcDNA3.1-FlagHA (a kind gift from Dr S. Rouquier, Toulouse, France) with murine phosphoglycerate kinase (PGK) promoter. Sanger sequencing confirmed all construct sequences.

### Cell culture and transfections

The 786-O kidney cancer cell line, from the ATCC (LGC Standards), was cultured in RPMI-1640 (Gibco^™^ - Life Technologies) supplemented with 10% fetal calf serum and 1% penicillin/streptomycin at 37°C with 5% CO_2_. Stable cell lines were generated by transfecting 786-O cells with pcDNA3.1-*P*_PGK_-Flag-HA-VHL_213_ or pcDNA3.1-*P*_PGK_-Flag-HA-VHL_172_ using JetPRIME (PolyPlus, Ozyme) and selected with 500μg/ml G418 (Gibco™). *VHL* knockdown experiments were performed by transfecting 75 nM siRNA against *VHL* variant 2 or a control siRNA [3] using JetPRIME for 72 hours. Cells were then harvested for western blotting and RT-PCR analysis.

### RNA extraction and RT-qPCR analysis

Total RNA was extracted from cells using the Nucleospin RNA reagent kit (Macherey-Nagel). cDNAs were synthesized from 1.5 μg (or 0.4 μg from spheroids) total RNA using random hexamer primers and M-MLV reverse transcriptase (Promega). All primers used for quantitative PCR (qPCR) are summarized in Supplementary Tables 2 and 3. PCR reactions were carried out using the GoTaq Flexi DNA Polymerase kit (Promega) and qPCR was performed as previously described [43].

### Protein extraction and immunoblotting

Cells were lysed in extraction buffer (EB) or in RIPA buffer [3], or directly in SDS-PAGE loading buffer. Equal amounts of proteins were separated by SDS-PAGE. Membranes were probed with antibodies against VHL (1:1,000; clone JD1956), HA (1:4,000; Roche), Flag (1:2,000; Sigma-Aldrich), β-tubulin (1:2,000; Sigma-Aldrich), cyclin D1 (1:500; Cell Signaling Technology), cullin-2 (1:450; Invitrogen™, Life Technologies), elongin-C (1:1,000; Bio Legend), HIF2-α (1:500; Novus Biologicals). Immune complexes were detected as described [3].

### Immunoprecipitation assays

Total protein extracts (250 μg/sample) in EB buffer were incubated with anti-Flag antibody (Sigma-Aldrich). Then 15μl of Affi-Prep® Protein A (Bio-Rad) were incubated with the mixture. Bound fractions were solubilized in SDS-PAGE loading buffer and analysed as described in [3].

### Zymography assay

Twenty μg of total protein extracts were separated in denaturing and non-reducing conditions on 8% polyacrylamide gels containing 1mg/ml gelatin type B (Sigma) or 0.5 mg/ml collagen type I (Sigma). Gels were washed twice with 2.5% Triton® X-100 for 20 min to eliminate SDS. After washes in buffer (50mM Tris pH8.0, 150mM NaCl, 5mM CaCl_2_, 2μM ZnCl_2_), gels were incubated in activation buffer at 37°C for 16 hours, and then stained with Coomassie® Blue.

### Immunofluorescence

Cells were cultured on coverslips for 48 hours and fixed with 3.7% paraformaldehyde. Cells were processed for immunocytochemistry as previously described [44] and stained with the rat anti-HA antibody (1/50 in PBS -1% BSA; Roche), followed by the secondary Alexa Fluor®647-conjugated anti-rat secondary antibody (1:1000; Abcam). Images were analysed with the ImageJ software (NIH).

### Immunohistochemistry

Four-μm sections of formalin-fixed, paraffin-embedded tumor samples were processed for immunohistochemistry, as previously described [16]. The anti-HA antibody (1:200; Roche) binding was revealed with horseradish peroxidase (HRP)-labelled polymer conjugated to secondary antibodies (Envision™ + Dual Link System-HRP, DAKO) and diaminobenzidine as chromogen (Sigma-Aldrich). CD31 immunostaining (1:25; Clinisciences) was performed using an automated slide staining system (Discovery XT -Ventana Medical Systems). Whole slide image acquisition was performed using a Nanozoomer 2.0-HT and the NDP.view2 viewing software (Hamamatsu).

### Proliferation assay

Cells were seeded in 96-well plates (500 cells/well) and cell proliferation was analyzed after 0, 2, 3, 4 or 5 days of culture. The PrestoBlue™ reagent (Invitrogen) was added according to the manufacturer's recommendation and fluorescence was quantified using a microplate reader (FLUOstar Omega – BMG Labtech).

### Wound healing assay

Cells were cultured at 5% CO_2_ at 37°C until confluence and then a scratch was performed manually. Cells were treated or not with 10μg/ml mitomycin C (Sigma-Aldrich). Cell migration was then recorded every hour for 24 hours using an Axiovert 200M microscope equipped with a LD Plan-Neofluar 20×/0.4 Ph2 lens and an AxioCam MRm camera under the control of the ZEN 2012 software (Zeiss). The wound area was measured using the SimplePCI6 software (Hamamatsu).

### Spheroid formation

5,000 cells were plated in medium in a well of a 96-well plate with round bottom previously coated with Poly(2-hydroxyethyl metacrylate) (Sigma) and incubated for 4 days to allow spheroid formation. The spheroids were analysed by microscopy (DMIRB- Leica) and the size calculated using Image J software.

### *In vivo* xenograft experiments

Five-week-old nude mice (BALB/cAnNRj-Foxn1nu/Foxn1nu; Janvier Laboratories) were subcutaneously injected with 786-O-pVHL_172_ cells on the left flank and with 786-O or 786-O-pVHL_213_ cells on the right flank (1.10^7^ cells/injection; five animals/group). Tumor size was monitored using an electronic calliper twice per week. Tumor volume was determined according to the equation: Vol. = length x width x thickness x 0.5236. Mice were finally euthanized and tumors harvested and cut in two pieces (one snap-frozen and the other formalin-fixed). The experimental protocol complied with the institution's guidelines for animal welfare and was approved by the Ethics Committee for Animal Experimentation of the French Ministry for Higher Education and Scientific Research (agreement # 2015072410433840 v2).

### Statistical analyses

Statistical analyses were performed with the R-studio software. Statistical significance was assessed using unpaired Student t, Kruskal-Wallis, Mann-Whitney and ANOVA tests. P < 0.05 was considered significant.

## SUPPLEMENTARY MATERIALS FIGURES AND TABLES




