# Supplementary material for: The pVHL172 isoform is not a tumor suppressor and up-regulates a subset of pro-tumorigenic genes including TGFB1 and MMP13
Source: Oncotarget. 2017 Jun 6;8(44):75989–6002. doi: 10.18632/oncotarget.18376 (PMC5652680; doi:10.18632/oncotarget.18376)
Supplement: Supplementary file 1 [file oncotarget-08-75989-s001.pdf]

The pVHL$_{172}$ isoform is not a tumor suppressor and up-regulates a subset of pro-tumorigenic genes including *TGFB1* and *MMP13*

SUPPLEMENTARY FIGURES AND TABLES

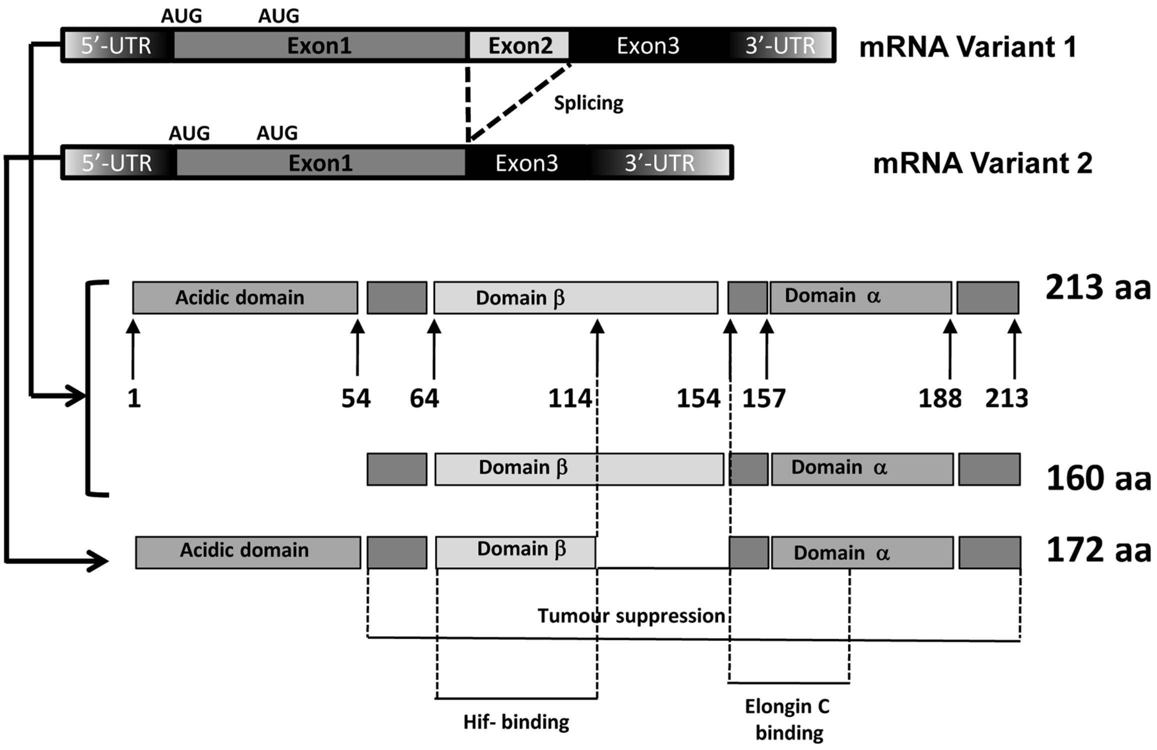

Supplementary Figure 1: Schema of the transcription and translation of the *vhl* gene.

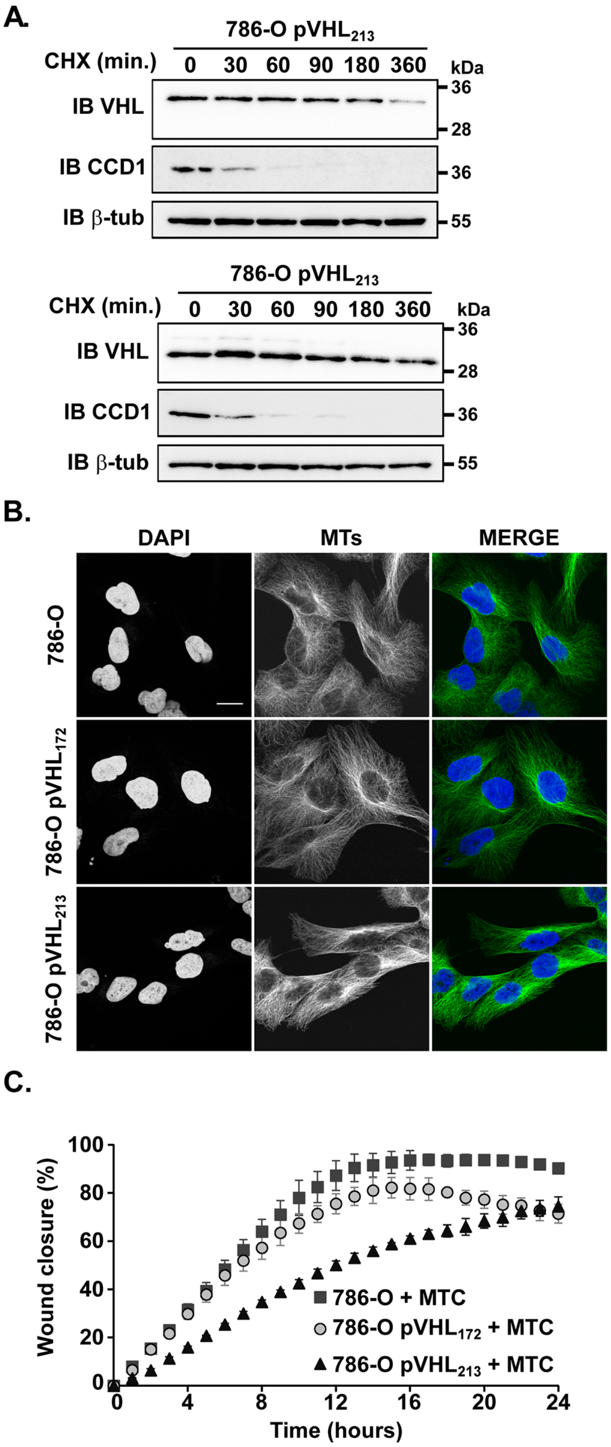

Supplementary Figure 2: (A) pVHL expression and stability in the 786-O-pVHL$_{172}$ cell line. Time-course of pVHL and cyclin-D1 (CCD1) expression in 786-O-pVHL$_{213}$ and 786-O-pVHL$_{172}$ cells was assessed by western blot analysis at different time points after addition of 50µg/ml cycloheximide (CHX). β-tubulin was used as loading control. Molecular weights are indicated on the right (kDa). **(B)** Tubulin staining, analyzed with an anti-tubulin in 786-O (upper panels), 786-O-pVHL$_{172}$ (middle panels) and 786-O-pVHL$_{213}$ cells (lower panels). Nuclei were stained with DAPI (scale bar: 25µm). **(C)** Wound healing assay in 786-O, 786-O-pVHL$_{172}$ and 786-O-pVHL$_{213}$-cells cultured in the presence of mitomycin C (MTC). Results are expressed as the percentage of wound closure at different time points (mean ± s.d. of three independent samples).

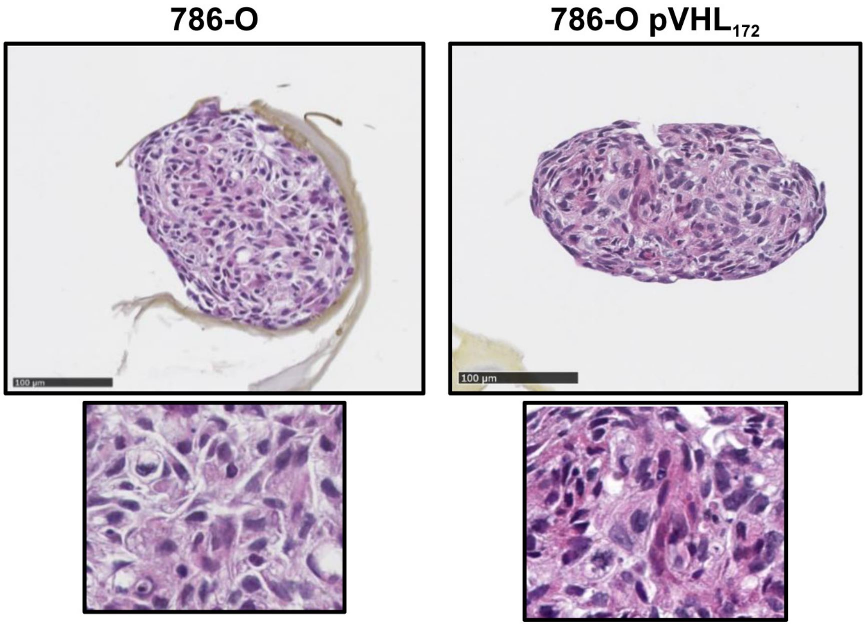

Supplementary Figure 3: HSE staining in 786-O and 786-O-pVHL$_{172}$ spheroids (scale bar: 100µm). A 20X magnification is shown in the lower panels (scale bar: 20 µm).

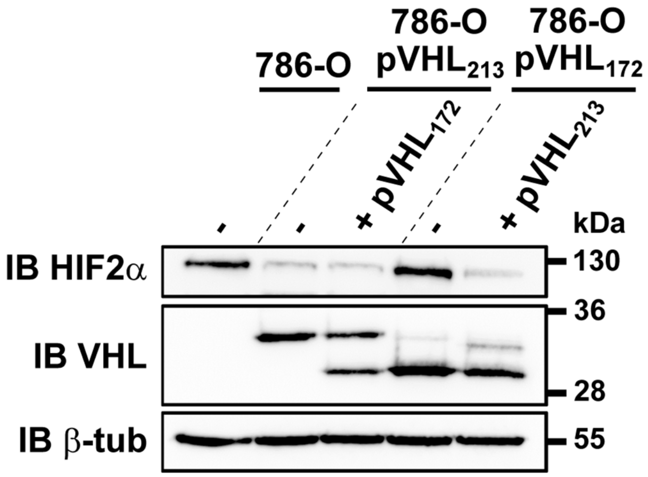

Supplementary Figure 4: HIF-2α expression level in 786-O, 786-O-pVHL$_{213}$ and 786-O-pVHL$_{172}$-cells transiently transfected (+) or not (-) with pVHL$_{172}$- or pVHL$_{213}$-expressing plasmids. β-tubulin was used as loading control.

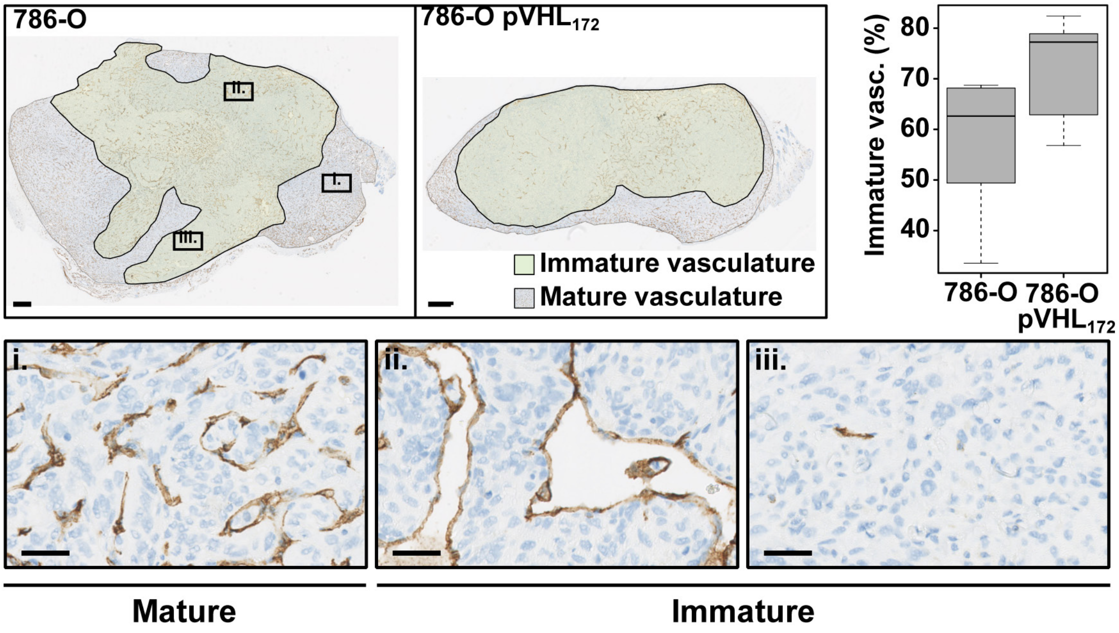

Supplementary Figure 5: Mature and immature vasculature areas were delimited following CD31-staining of 786-O (left panel) and pVHL$_{172}$-cell derived (right panel) tumor sections (scale bar: 200µm). Insets in a representative 786-O-cell derived tumor section show mature vasculature with short vessels displaying few ramifications and high vascular density (i), and immature vasculature characterized by large and ramified vessels (ii) or by low vascular density (iii) (scale bar: 100µm). The box plot shows the percentage of immature vasculature regions in 786-O and 786-O-pVHL$_{172}$ cell-derived tumors relative to the whole tumor area.

Supplementary Table 1: Size of the tumors

See Supplementary File 1

Supplementary Table 2: PCR primers used in the study

Human mRNA	Primer sequence	Amplicon size (bp)
FlagHAVHL	Fw: 5'-ATGGATTACAAGGATGACGACG-3'<br>Rev: 5'-AGATCTTCGTAGAGCGACCTGA-3'	635 (*) ; 498 (**)

Fw: forward primer; Rev: reverse primer; * in cells expressing FlagHA-VHL_{213}, ** in cells expressing FlagHA-VHL_{172}

Supplementary Table 3: PCR primers used in the study (in qPCR analyses)

Human mRNA	Primer sequence	Amplicon size (bp)
GAPDH	Fw: 5'-AATGACCCCTTCATTGACCTC-3'<br>Rev: 5'-TTCCATTGATGACAAGCTTCC-3'	115
MMP1	Fw: 5'-AGGTCTCTGAGGGTCAAGCA-3'<br>Rev: 5'-CTGGTTGAAAAGCATGAGCA-3'	111
MMP2	Fw: 5'-ATGACAGCTGCACCACTGAG-3'<br>Rev: 5'-AGTTCCCACCAACAGTGGAC-3'	126
MMP13	Fw: 5'-TGGTCCGATGTAACTCCTCTG-3'<br>Rev: 5'-TAGAAGTCGCCATGCTCCTTA-3'	95
RPLP0	Fw: 5'-CTGTTGCATCAGTACCCCATT-3'<br>Rev: 5'-CTTGACCTTTTCAGCAAGTGG-3'	101
TGFβ1	Fw: 5'-AACACATCAGAGCTCCGAGAA-3'<br>Rev: 5'-GAGGTATCGCCAGGAATTGTT-3'	141
